# The Reicher-Wheeler paradigm in word recognition research: a cautionary note on its actual contributions and published misconceptions

**DOI:** 10.3389/fpsyg.2024.1399237

**Published:** 2024-10-21

**Authors:** Timothy R. Jordan, Aziz M. Akkaya, Fatma Zehra Göçmüş, Aleynanur Kalan, Ebru Morgul, Kübra Önalan, Mercedes Kier Sheen

**Affiliations:** ^1^School of Psychology, Canadian University Dubai, Dubai, United Arab Emirates; ^2^Department of Psychology, Ibn Haldun University, Istanbul, Türkiye; ^3^Department of Psychology, Heriot-Watt University, Dubai, United Arab Emirates

**Keywords:** Reicher-Wheeler paradigm, word recognition, visual word perception, word superiority effects, misconceptions

## Abstract

The study of word recognition has been influenced greatly by findings obtained when visual stimuli are presented very briefly. Under these conditions, a great deal of evidence suggests that words are perceived better than nonwords, and even single letters, and it is generally accepted that these “word superiority effects” reflect the relative efficiency with which words are perceived. For more than 50 years, a key procedure for establishing these effects has been the Reicher-Wheeler Paradigm in which potentially confounding effects of non-perceptual guesswork are cleverly suppressed. More recently, however, the actual nature of the Reicher-Wheeler paradigm and its contribution to research have become misrepresented in a range of publications, and its use in experiments has been confused and conflated with other, less sophisticated procedures. In this article we describe the actual contributions made by the Reicher-Wheeler Paradigm to word recognition research and show examples of how these important contributions have been misunderstood and misconceived in experiments reported in the recent literature.

## Introduction

Identifying the intricate perceptual processes involved in recognizing visually presented words has been a focus of empirical research for almost 140 years (e.g., Cattell, 1885, 1886; Erdmann and Dodge, 1898; Huey, 1908; Pillsbury, 1897; Wundt, 1900a,b; Zeitler, 1900). During this time, information about these perceptual processes has frequently been sought by investigating the relative perceptibility of words and other linguistic items (typically, nonwords and single letters) when these stimuli are presented for very brief durations (e.g., 50 ms). Under these conditions, a particularly fascinating finding is that words are easier to identify than nonwords, and even single letters, and reports of this phenomenon date from some of the earliest experiments in the history of psychology (Cattell, 1886; Erdmann and Dodge, 1898; see also Huey, 1908).

At first sight, these early findings indicated that words are more perceptible than other types of linguistic stimuli, and such an advantage for the perceptual processing of words would provide important clues to how words are normally read. However, for many years after these early experiments were conducted, interpretations of these findings based on the perceptual processing of stimuli were confounded by the possibility that at least part of the advantage for words (and, perhaps, all of it) over other types of stimuli reflected the ability of participants to use sophisticated, non-perceptual guesswork to artifactually boost word performance. As a result, the phenomenon was not necessarily a demonstration of the relative *perceptibility* of each type of stimulus but merely a demonstration that the identities of words could be guessed better than other types of stimuli, which would be a much less illuminating finding for understanding the perceptual processes involved.

To explain this problem more fully, consider the capabilities of a word (e.g., TABLE) and a nonword (e.g., TJQPZ, often called illegal nonwords) to be fully identified as the stimulus they each represent. Although both types of stimulus contain all the perceptual information required for their complete, accurate identification when viewing time is unlimited, brief presentations (e.g., lasting 50 ms) are designed to limit perceptual processing, and disruptions in the perceptual information encoded from stimuli will occur frequently when stimuli are displayed this briefly. Thus, when participants are required to identify a word or a nonword that has been presented briefly, a substantive part of this identification process may be the use of non-perceptual guesswork to help determine which components of words and nonwords (e.g., letters, groups of letters) can fit the incomplete perceptual input provided by the stimulus itself. As a result, this artifactual process may be used to help complete the identity of the whole stimulus, irrespective of whether it was a word or nonword. But if this were occurring, knowledge of the words that exist in the language and how they are constructed (e.g., the letters and letter groups they contain) may help determine which components are missing from the perceptual input provided by a briefly presented word stimulus and so help its non-perceptual completion. But nonwords, by comparison, do not exist in the language, and so are not constrained by the same linguistic knowledge. Indeed, although this distinction is most clear with illegal nonwords (like TJQPZ), the same logic may be applied even to nonwords that share similarities with real words (often called pseudowords, like TUCLE) but which are still not real words. As a result, incomplete perception of briefly presented nonwords does not have the same opportunities for artifactual non-perceptual completion as words. In this way, even if words and nonwords were actually *equally* perceptible when presented briefly, an advantage for words could be produced because perceptual omissions for words are more likely to be filled artifactually by knowing the identities and structures of words in the language; nonwords by their nature do not provide the same opportunities. Similar arguments also exist for single letter displays, which also cannot benefit from knowing the identities and structures of words in the language. Here, the crucial point is that identification of a single letter stimulus requires perception of that stimulus alone and no other information in the display can provide cues to the letter’s identity (i.e., there is no other information about the identity of a single letter stimulus in single letter displays). In sum, enormous achievements were made by ingenious experiments conducted at the very start of empirical research into visual word perception. But it remained a possibility that performance with word stimuli in these earlier studies was enhanced artifactually by non-perceptual guesswork and that this non-perceptual enhancement was not available for other types of linguistic stimuli (for further discussions, see Henderson, 1982; Johnston, 1978). Thus, although non-perceptual guesswork may be useful in everyday life (for example, when reading print of visually poor quality), research intended to reveal the perceptual processing of different linguistic stimuli may be contaminated by such non-perceptual distortions.

## Actual contributions of the Reicher-Wheeler paradigm

For many years, the absence of a paradigm that suppresses influences of non-perceptual guesswork on performance remained a major obstacle to assessing the relative perceptibility of words and other linguistic stimuli. But eventually such a paradigm was developed, and this became known as the *Reicher-Wheeler Paradigm* (hereafter, R-WP) after it was first reported by Reicher (1969) and Wheeler (1970). Using the R-WP, the perceptibility of stimuli is limited by using brief presentations (as in previous studies) but the fundamental difference compared to earlier research is that each stimulus is followed by two alternative letters and participants must decide which one of these letters occurred at a specified location in the stimulus itself. Of particular importance is that both letters are consistent with the rest of the stimulus and so selecting the correct response cannot be assisted by non-perceptual guesswork. For example, consider comparing performance for words (e.g., TABLE) and nonwords (e.g., TJQPZ). Following a brief presentation of the word TABLE, perceptibility of the stimulus may be assessed by requiring participants to choose between (in this example) the letters T and C in the initial location. Crucially, when a word has been shown, *both* letters form a word with the remainder of the stimulus (TABLE/CABLE) and so selecting the correct letter cannot be assisted non-perceptually by using knowledge about what words exist in the language, how they are spelled, and how each letter fits information from any other part of the stimulus. Moreover, the same technique can be used to assess perception of nonwords, where the brief presentation of TJQPZ would also be followed by the same letters (T and C) in the initial location, and where both letters form a nonword with the remainder of the stimulus. In this way, the letter presented in a stimulus and its forced-choice alternative are identical for word and nonword displays, and the accuracy of responses for both stimulus types cannot be enhanced by non-perceptual deduction as both alternatives are plausible for each stimulus type (for further discussion of this logic, see Johnston, 1978; Reicher, 1969; Wheeler, 1970).

As a consequence, when using the R-WP, differences in performance between words and nonwords can reasonably be attributed to differences in the *perception* of each stimulus type (word, nonword) rather than to differences in the effectiveness of non-perceptual guesswork. The same procedure can also be used for matched single letter stimuli (in this example, T), where responses to the same alternatives (T and C) indicate the perception of the same letter (T) in isolation. Following the findings of Johnston (1978; see also Jordan et al., 2000), the paradigm also appears to rule out effects of guesswork not only during the overt selection of a response but at *any* stage of processing (e.g., even during early perceptual processing where, conceivably, covert influences based on partial word information may exist). Indeed, Johnston also found that, although the R-WP produced substantial word superiority over illegal nonwords and single letters, free report of the same critical letters in the same stimuli (but without forced-choice alternatives to suppress effects of non-perceptual guesswork) produced considerably greater word superiority effects, over both nonwords and single letters. This increase in word superiority is consistent with the view that the R-WP suppresses influences of non-perceptual guesswork that may otherwise selectively (and artifactually) inflate performance with words (see also discussions by Estes, 1975).

Consequently, the R-WP appears to be well-suited to investigating the perceptibility of words and other types of linguistic stimuli without contamination from non-perceptual artifacts. So, it is hardly surprising that, for many years, findings obtained using the R-WP have greatly influenced theoretical approaches to word recognition where perception of words, rather than influences of guesswork, has been the focus. These theoretical approaches include the Interactive-Activation Model of word perception (e.g., McClelland and Rumelhart, 1981; Rumelhart and McClelland, 1982), and other accounts of word recognition (e.g., Carr and Pollatsek, 1985; Coltheart et al., 2001; Jacobs and Grainger, 2005; Paap et al., 1982). Indeed, experiments using the R-WP have inspired insights into a variety of established word recognition phenomena (e.g., Jordan and Bevan, 1994, 1996; Jordan and De Bruijn, 1993; Jordan and Patching, 2004; Jordan et al., 2000; Jordan et al., 2003. Jordan and Thomas, 2002).

## Misconceptions of the Reicher-Wheeler paradigm in the literature

The benefits of using the R-WP for word recognition research have been understood and appreciated by innumerable researchers since the inception of the paradigm more than 50 years ago (some indicative examples are Carr et al., 1978; Carr et al., 1976; Carr and Pollatsek, 1985; Johnston, 1978; Johnston and McClelland, 1973, 1974; Jordan and Bevan, 1994, 1996; Jordan and De Bruijn, 1993; Jordan and Kalan, 2024; Jordan et al., 2000; Jordan and Thomas, 2002; McClelland and Johnston, 1977; McClelland and Rumelhart, 1981; Reuter-Lorenz and Baynes, 1992; Rumelhart and McClelland, 1982). However, in recent years, these benefits appear to have been misconceived by some researchers, and claims have been made in the literature about the characteristics and use of the R-WP that are fundamentally inaccurate. As a result, these errors obscure the true nature of the findings that were produced by the experiments reported in these more recent studies and are likely to misinform the conclusions drawn by the authors and by readers of the articles produced. Let us now look at examples of the errors that have been made when using and reporting the R-WP.

Most recently, an article by Marzouki et al. (2022) claimed to have used the R-WP to investigate the relative perceptibility of briefly presented Arabic words, pseudowords, and nonwords. As we have explained, a crucial component of the R-WP (and perhaps its core genius) is the use of two equally plausible letters shown after each stimulus so that stimulus perception can be assessed without contamination from non-perceptual guesswork. Most importantly, as both alternative letters are consistent with the remainder of each stimulus, correct selection cannot be enhanced non-perceptually especially when the stimulus is a word. But instead of using the R-WP, and despite claiming repeatedly that the R-WP was used, Marzouki et al. used an older and more basic technique (often called the *probe task* or *bar marker task*, e.g., Averbach and Coriell, 1961) in which a letter in the stimulus is simply highlighted for report (usually by an arrow or some other cue) after the stimulus has been presented; in the study by Marzouki et al., two horizontal lines were shown above and below the position of the letter required for report. But this probe procedure used by Marzouki et al. is clearly vulnerable to influences of non-perceptual guesswork that inspired the invention of the R-WP; namely, that without the precautions offered by two carefully chosen alternative letters, report of a letter in a linguistic stimulus (word, pseudoword, nonword) can be enhanced non-perceptually and this artifactual enhancement may be greatest for words. Indeed, in the study by Marzouki et al., letters were reported more accurately in words than in pseudowords or nonwords, and more accurately in pseudowords than in nonwords. This pattern of effects is consistent with the influences of non-perceptual guesswork that were first addressed more than 50 years ago by the R-WP, and which inspired this shift in procedure when investigating the perceptibility of different types of linguistic stimuli. Accordingly, the study by Marzouki et al. *did not* use the R-WP and so it remains to be seen if the findings reported would have been observed if their study had actually used the R-WP. However, it is also worth mentioning that the article by Marzouki et al. did report a previous published study of the relative perceptibility of Arabic words, pseudowords, and nonwords that had already revealed word superiority effects in Arabic using the R-WP (Jordan et al., 2010). In particular, the procedures used in this earlier experiment were in accord with the key requirement of the R-WP that two equally plausible letters are shown after each stimulus is presented, and the findings had shown clear word superiority effects under these stringent experimental conditions. This paradox of citing a previous study that had actually used the R-WP to show word superiority effects in Arabic, and then inaccurately reporting that a new study had used the R-WP when it had not, serves to illustrate how the crucial contributions of the R-WP to word recognition research can be easily misunderstood.

But Marzouki et al. (2022) are not alone in their misconception of the key role of the forced-choice procedure used in the R-WP and which sets this paradigm apart. Mok (2009) describes the merits of using the R-WP to investigate the relative perceptibility of two-character Chinese words and nonwords. But Mok did not use the two-alternative forced choice procedure crucial for the R-WP. Instead, each two-character stimulus (word or nonword) was briefly presented (for 40–80 ms) during which time one of the characters was underlined on the screen. This underline persisted for 500 ms and participants had to write down the character concerned. But, like the task used by Marzouki et al. (2022), the task used by Mok is also vulnerable to the influences of non-perceptual guesswork that inspired the invention of the R-WP. Specifically, without the precautions provided by the R-WP, identification of a target character can be enhanced non-perceptually by other aspects of the stimulus and this artifactual enhancement may be greatest for words. In fairness, Mok states very reasonably in the Procedure section that modifications were made to the Reicher-Wheeler paradigm because of the relative difficulty of finding matching choice alternatives with Chinese materials (p. 1057). But unfortunately, these modifications removed a component of the R-WP that is so crucial for determining the relative perceptibility of different types of stimuli that the procedure was no longer the R-WP.

But although the procedures used in the studies by Marzouki et al. (2022) and Mok (2009) were *obviously* not the R-WP, other articles describing the use of the R-WP have shown less obvious, but still crucial, deviations from the correct implementation of this paradigm. For example, Chen et al. (2018) report using the R-WP to investigate the relative perceptibility of two-character Chinese words and nonwords. Each stimulus was presented briefly, followed by two Chinese characters (called a “probe”), one of which had been present in the stimulus and one of which had not. Participants simply had to choose which of these characters had been in the stimulus. Importantly, both characters in each probe were consistent with the stimulus such that each character formed a word with the remainder of the stimulus when a word had been shown and each formed a nonword with the remainder of the stimulus when a nonword had been shown. This procedure is analogous to the more usual R-WP procedure in which each stimulus is followed by a choice between two letters that are both consistent with the stimulus just presented, word or nonword. However, letters vary in their individual perceptibility (e.g., Bouma, 1971; Grainger et al., 2008; Pelli et al., 2006; see also Dyson, 2013), and it is normal practice in the R-WP to use the same two-letter pairs in the forced choice task (corresponding to the two-character probes in the Chen et al. study) for all types of stimuli in an experiment (e.g., words, nonwords, single letters). In this way, differences in performance across different stimulus types can reasonably be attributed to the different stimulus conditions and not to differences in the letter alternatives that were used. But Chen et al. used different two-character probes for word and nonword stimuli in their study, although Chinese characters also differ in their individual perceptibility (e.g., Yang and Wang, 2018; see also Tsao and Wang, 1983). Indeed, both characters in each probe differed between word and nonword conditions, and so a substantial confound existed between the identities of probe characters and stimulus type, with unknown consequences for performance. Chen et al. were aware of these problems and attempted to match character frequency and number of strokes for the different characters used across words and nonwords. But, notwithstanding these laudable efforts, the actual relative perceptibility of the different characters that were used is unknown. Nevertheless, the article concludes that, using the R-WP, “the experiments found that a character was better recognized when it was part of a word than when it was part of a nonword.” (Discussion, p.13). In a sense, this statement is true, but the characters were not the same characters, and this description of the methodology and the findings it produced may be misleading to readers. Comparable failures to match forced-choice alternatives across stimulus conditions when claiming to use the R-WP can also be found with letter-based stimuli and letter alternatives (Casaponsa and Duñabeitia, 2016; Laszlo and Federmeier, 2007), and across languages as diverse as Korean (Chen et al., 2017) and Croatian (Matić et al., 2018).

## Discussion

The findings of the studies we have described remain to be determined under true R-WP conditions and so the validity of their interpretations is currently unclear. However, these published examples are not offered as exhaustive and, indeed, the absence of information in many other studies claiming to have used the R-WP may well obscure errors that were also made in implementing the paradigm correctly. So, it seems very likely that more misconceptions of the R-WP already exist in the literature and that more will occur in the future. But the examples presented here should, we hope, provide a timely alert for researchers who are considering using the R-WP in experiments, for readers of articles that report using the R-WP in experiments, and for reviewers and editors assessing, for publication, manuscripts that report using the R-WP in experiments.

As research into word recognition evolves, other techniques are likely to be developed to help determine the perceptual processes used for identifying words and other types of linguistic stimuli, without confounds from non-perceptual influences. For example, fMRI has already been applied to investigations of the effects of word context on cortical activity (e.g., Heilbron et al., 2020), and findings suggest that word context enhances letter representations. In addition, other studies suggest that ERPs are modulated by the lexical status of stimuli around 200 ms after stimulus onset and that visual word form representations constrain letter identification at a prelexical level (Martin et al., 2006). But, for the foreseeable future, the use of cleverly constructed linguistic stimuli and the logic of the R-WP are likely to be crucial, and comparisons in performance across different paradigms (e.g., R-WP vs. simple probed report) will be valuable for identifying the involvement of perceptual and non-perceptual processes at the neural level. Indeed, knowing the effects produced by different paradigms on identifying linguistic stimuli should facilitate accurate interpretations of the findings from imaging research; without knowing the effects that different paradigms exert on the use of perceptual and non-perceptual processes for word recognition, attempts to accurately identify the neural *perceptual* processing of words may be confounded by neural *non-perceptual* processing. So, for some time to come, gaining new information about perception of words may benefit from the correct use and understanding of the Reicher-Wheeler Paradigm.
